# Supplementary material for: Multi‐dataset identification of innovative feature genes and molecular mechanisms in keratoconus
Source: J Cell Mol Med. 2024 Sep 19;28(18):e70079. doi: 10.1111/jcmm.70079 (PMC11412914; doi:10.1111/jcmm.70079)
Supplement: Supplementary file 2 — Material S2. [file JCMM-28-e70079-s002.zip › 7.GSEA analysis/4.MYRF/KEGG_HYPERTROPHIC_CARDIOMYOPATHY_HCM.html]

Details for gene set KEGG\_HYPERTROPHIC\_CARDIOMYOPATHY\_HCM[GSEA]

|  || Dataset | MYRF.group.cls#h\_versus\_l |
| Phenotype | group.cls#h\_versus\_l |
| Upregulated in class | l |
| GeneSet | KEGG\_HYPERTROPHIC\_CARDIOMYOPATHY\_HCM |
| Enrichment Score (ES) | -0.42501527 |
| Normalized Enrichment Score (NES) | -1.4706398 |
| Nominal p-value | 0.020242915 |
| FDR q-value | 0.21227805 |
| FWER p-Value | 0.801 |
Table: GSEA Results Summary

  

Fig 1: Enrichment plot: KEGG\_HYPERTROPHIC\_CARDIOMYOPATHY\_HCM      
 Profile of the Running ES Score & Positions of GeneSet Members on the Rank Ordered List

  

| SYMBOL | TITLE | RANK IN GENE LIST | RANK METRIC SCORE | RUNNING ES | CORE ENRICHMENT || 1 | TNNT2 | na | 75 | 0.896 | 0.0508 | No |
| 2 | CACNG8 | na | 755 | 0.506 | 0.0610 | No |
| 3 | TNNI3 | na | 1269 | 0.424 | 0.0713 | No |
| 4 | CACNG5 | na | 1534 | 0.398 | 0.0872 | No |
| 5 | ITGA4 | na | 1618 | 0.389 | 0.1077 | No |
| 6 | TTN | na | 2608 | 0.317 | 0.0979 | No |
| 7 | ACTC1 | na | 3638 | 0.266 | 0.0840 | No |
| 8 | DAG1 | na | 4906 | 0.225 | 0.0607 | No |
| 9 | MYBPC3 | na | 5513 | 0.207 | 0.0554 | No |
| 10 | CACNB1 | na | 5538 | 0.206 | 0.0669 | No |
| 11 | ITGA9 | na | 5848 | 0.197 | 0.0697 | No |
| 12 | TNNC1 | na | 8243 | 0.132 | 0.0084 | No |
| 13 | IGF1 | na | 8743 | 0.121 | 0.0012 | No |
| 14 | EMD | na | 9064 | 0.115 | -0.0013 | No |
| 15 | ITGB7 | na | 9321 | 0.109 | -0.0023 | No |
| 16 | PRKAG2 | na | 9896 | 0.098 | -0.0131 | No |
| 17 | TPM4 | na | 10259 | 0.091 | -0.0181 | No |
| 18 | CACNB2 | na | 10834 | 0.081 | -0.0299 | No |
| 19 | MYH6 | na | 11121 | 0.076 | -0.0337 | No |
| 20 | CACNA2D3 | na | 11412 | 0.072 | -0.0378 | No |
| 21 | DES | na | 11440 | 0.071 | -0.0343 | No |
| 22 | CACNA1C | na | 11956 | 0.064 | -0.0454 | No |
| 23 | TPM1 | na | 12030 | 0.063 | -0.0438 | No |
| 24 | ITGB6 | na | 12239 | 0.060 | -0.0463 | No |
| 25 | ACTB | na | 12485 | 0.057 | -0.0500 | No |
| 26 | MYH7 | na | 12644 | 0.055 | -0.0513 | No |
| 27 | PRKAG1 | na | 13773 | 0.041 | -0.0815 | No |
| 28 | CACNA1D | na | 13802 | 0.040 | -0.0799 | No |
| 29 | ATP2A2 | na | 14195 | 0.036 | -0.0891 | No |
| 30 | PRKAG3 | na | 14342 | 0.034 | -0.0913 | No |
| 31 | DMD | na | 14406 | 0.033 | -0.0912 | No |
| 32 | ACE | na | 14534 | 0.032 | -0.0929 | No |
| 33 | CACNG7 | na | 16278 | 0.015 | -0.1424 | No |
| 34 | PRKAA1 | na | 17163 | 0.007 | -0.1675 | No |
| 35 | TPM2 | na | 17462 | 0.004 | -0.1758 | No |
| 36 | CACNG1 | na | 17979 | -0.000 | -0.1907 | No |
| 37 | CACNG2 | na | 18631 | -0.007 | -0.2091 | No |
| 38 | ITGB4 | na | 19208 | -0.013 | -0.2250 | No |
| 39 | LMNA | na | 20403 | -0.027 | -0.2578 | No |
| 40 | TPM3 | na | 20434 | -0.027 | -0.2571 | No |
| 41 | MYL3 | na | 20790 | -0.031 | -0.2656 | No |
| 42 | PRKAB2 | na | 21337 | -0.037 | -0.2791 | No |
| 43 | TGFB2 | na | 22177 | -0.048 | -0.3005 | No |
| 44 | ACTG1 | na | 22317 | -0.050 | -0.3016 | No |
| 45 | LAMA2 | na | 23159 | -0.062 | -0.3222 | No |
| 46 | ITGA3 | na | 23712 | -0.071 | -0.3339 | No |
| 47 | ITGB5 | na | 23730 | -0.071 | -0.3302 | No |
| 48 | ITGA2 | na | 25150 | -0.096 | -0.3655 | No |
| 49 | ITGA2B | na | 25913 | -0.111 | -0.3809 | No |
| 50 | CACNB3 | na | 26225 | -0.118 | -0.3829 | No |
| 51 | PRKAB1 | na | 26497 | -0.123 | -0.3835 | No |
| 52 | ITGAV | na | 26862 | -0.132 | -0.3862 | No |
| 53 | SGCA | na | 27650 | -0.152 | -0.4000 | No |
| 54 | ITGA11 | na | 28180 | -0.166 | -0.4054 | No |
| 55 | SGCG | na | 28859 | -0.187 | -0.4140 | Yes |
| 56 | CACNA1F | na | 28862 | -0.187 | -0.4030 | Yes |
| 57 | SGCD | na | 29214 | -0.197 | -0.4015 | Yes |
| 58 | ITGA7 | na | 29295 | -0.200 | -0.3920 | Yes |
| 59 | ITGA8 | na | 29346 | -0.201 | -0.3816 | Yes |
| 60 | ITGA6 | na | 29414 | -0.203 | -0.3715 | Yes |
| 61 | PRKAA2 | na | 29444 | -0.204 | -0.3603 | Yes |
| 62 | CACNG6 | na | 29465 | -0.205 | -0.3488 | Yes |
| 63 | ITGB3 | na | 29529 | -0.207 | -0.3384 | Yes |
| 64 | ITGB1 | na | 29873 | -0.219 | -0.3353 | Yes |
| 65 | RYR2 | na | 30209 | -0.231 | -0.3314 | Yes |
| 66 | MYL2 | na | 30601 | -0.246 | -0.3281 | Yes |
| 67 | ITGB8 | na | 30931 | -0.260 | -0.3223 | Yes |
| 68 | CACNG4 | na | 31907 | -0.312 | -0.3320 | Yes |
| 69 | CACNA2D2 | na | 32005 | -0.317 | -0.3161 | Yes |
| 70 | ITGA10 | na | 32096 | -0.323 | -0.2996 | Yes |
| 71 | SGCB | na | 32475 | -0.349 | -0.2899 | Yes |
| 72 | CACNA2D4 | na | 32488 | -0.350 | -0.2696 | Yes |
| 73 | TGFB1 | na | 32868 | -0.377 | -0.2582 | Yes |
| 74 | TGFB3 | na | 33024 | -0.389 | -0.2397 | Yes |
| 75 | CACNA1S | na | 33196 | -0.406 | -0.2206 | Yes |
| 76 | SLC8A1 | na | 33227 | -0.410 | -0.1973 | Yes |
| 77 | IL6 | na | 33353 | -0.425 | -0.1758 | Yes |
| 78 | ITGA1 | na | 33768 | -0.482 | -0.1593 | Yes |
| 79 | CACNB4 | na | 34188 | -0.569 | -0.1378 | Yes |
| 80 | CACNG3 | na | 34232 | -0.580 | -0.1048 | Yes |
| 81 | TNF | na | 34328 | -0.609 | -0.0715 | Yes |
| 82 | ITGA5 | na | 34505 | -0.679 | -0.0365 | Yes |
| 83 | CACNA2D1 | na | 34582 | -0.726 | 0.0042 | Yes |
Table: GSEA details [plain text format]

  

Fig 2: KEGG\_HYPERTROPHIC\_CARDIOMYOPATHY\_HCM      
 Blue-Pink O' Gram in the Space of the Analyzed GeneSet

  

Fig 3: KEGG\_HYPERTROPHIC\_CARDIOMYOPATHY\_HCM: Random ES distribution      
 Gene set null distribution of ES for **KEGG\_HYPERTROPHIC\_CARDIOMYOPATHY\_HCM**
